# Composition Design and Property Investigation of Mold Fluxes for the Continuous Casting of Rare-Earth Weathering Steel

**DOI:** 10.3390/ma19112236

**Published:** 2026-05-25

**Authors:** Zhihong Liu, Yang Wang, Lijun Xu

**Affiliations:** 1 Zhongda National Engineering & Research Center of Continuous Casting Technology Co., Ltd., Beijing 100081, China; l_honly@163.com (Z.L.); ljxuah@sina.com (L.X.); 2 China Iron and Steel Research Institute Co., Ltd., Beijing 100081, China

**Keywords:** rare-earth weathering steel, mold flux, low-reactivity design, CaO–Al_2_O_3_-based flux, Ce_2_O_3_ absorption, thermodynamic modeling, viscosity, crystallization behavior

## Abstract

Conventional CaO–SiO_2_-based mold fluxes used in the continuous casting of rare-earth weathering steel are prone to severe slag–steel interfacial reactions, resulting in marked compositional changes and progressive property deterioration after rare-earth oxide pickup, which compromises lubrication stability and casting operability. In this study, a novel low-reactivity CaO–Al_2_O_3_-based mold flux was designed through phase-diagram-guided composition design, IMCT-based thermodynamic screening, and experimental investigation of melting, viscosity, and crystallization behavior. The originality of this work lies in establishing a design–validation framework for fluxes that remain stable after Ce_2_O_3_ absorption, rather than simply replacing the conventional CaO–SiO_2_ system. Validation against literature SiO_2_ activity data showed good trend consistency, supporting the use of the IMCT model as a semi-quantitative tool for composition screening. The results showed that CaO, Li_2_O, and Ce_2_O_3_ exhibited relatively high activities, whereas B_2_O_3_ showed extremely low activity. When the Ce_2_O_3_ content exceeded 5 wt.%, the viscosity remained about 0.30 Pa·s, and when the *w*(CaO)/*w*(Al_2_O_3_) ratio was higher than 1.6, it stabilized at about 0.25 Pa·s. The minimum melting temperature, 1122.6 °C, was obtained at 5 wt.% Ce_2_O_3_. Compared with a conventional CaO–SiO_2_-based flux, the developed flux showed similar initial processability but much better stability after absorbing 15 wt.% Ce_2_O_3_, with less severe deterioration in melting, viscosity, and crystallization behavior. These results provide scientific insight into mold-flux design under rare-earth oxide pickup conditions and practical guidance for improving the continuous casting of rare-earth weathering steels.

## 1. Introduction

Rare-earth (RE) steels, i.e., steels containing small additions of rare-earth elements, have attracted extensive attention because rare-earth elements can purify molten steel, modify non-metallic inclusions, and produce beneficial microalloying effects. As a result, even a small amount of rare-earth addition can significantly improve the strength, impact toughness, and corrosion resistance of steels, which has promoted their application in carbon steels, alloy steels, stainless steels, and, more recently, weathering steels [1]. Despite these advantages, the continuous casting of RE-bearing steels remains challenging because the high chemical activity of rare-earth elements can strongly disturb the steel/slag interfacial reactions and thereby affect mold-flux stability, lubrication, and heat transfer in the mold.

For conventional CaO–SiO_2_-based mold fluxes, severe interaction with rare-earth elements and rare-earth oxides is difficult to avoid during continuous casting. On the one hand, dissolved rare-earth elements in molten steel can react readily with reactive slag components, especially SiO_2_, causing significant slag compositional changes. On the other hand, rare-earth oxides generated in the steel can continuously enter the slag during casting, further altering the flux chemistry and its physicochemical properties [2]. These changes usually increase viscosity and crystallization tendency, impair slag consumption and lubrication, and destabilize mold heat transfer [3]. In practice, such instability may increase the risk of excessive slag-rim formation, unstable casting operation, surface longitudinal cracking, and deterioration of slab surface quality, which limits the wider application of RE-bearing steels in continuous casting [4,5].

Considerable efforts have been devoted to understanding the effects of rare-earth oxides on mold-flux behavior [6,7,8,9,10]. Previous studies have shown that rare-earth oxides can markedly influence melting temperature, viscosity, and crystallization behavior, and that the resulting trends depend strongly on slag basicity and composition. In parallel, CaO–Al_2_O_3_-based mold fluxes with Li_2_O and/or B_2_O_3_ additions have been investigated as low-reactivity alternatives to conventional silicate-based systems, mainly because they can reduce the melting temperature without relying on highly reactive SiO_2_. However, most previous studies have focused on the role of individual components or on the initial physicochemical properties of candidate fluxes, while comparatively less attention has been paid to the integrated design of mold fluxes for RE weathering steel under continuous rare-earth oxide pickup conditions. In particular, there is still a lack of clear understanding of how to design a low-reactivity flux system that can simultaneously maintain acceptable melting behavior, viscosity stability, and controllable crystallization behavior after absorbing substantial amounts of rare-earth oxides.

In view of the above issues, the present work aims to develop a dedicated low-reactivity mold flux for the continuous casting of rare-earth weathering steel based on the CaO–Al_2_O_3_–Li_2_O–B_2_O_3_–Ce_2_O_3_ system. The study combines phase-diagram-guided composition design, thermodynamic analysis based on the ion and molecule coexistence theory (IMCT), and experimental investigation of melting, viscosity, and crystallization behavior. The originality of this work lies not simply in replacing the CaO–SiO_2_ system with a CaO–Al_2_O_3_-based one, but in establishing an integrated design and validation framework for RE-steel mold fluxes that considers (i) reduced interfacial reactivity, (ii) physicochemical stability after Ce_2_O_3_ absorption, and (iii) the evolution of crystallization behavior compared with a conventional mold flux. By clarifying these relationships, this study is expected to provide both scientific insight into flux design under rare-earth oxide pickup conditions and practical guidance for improving lubrication stability, casting operability, and slab surface quality in the continuous casting of RE weathering steels.

## 2. Slag System Composition Design

**Figure 1:**
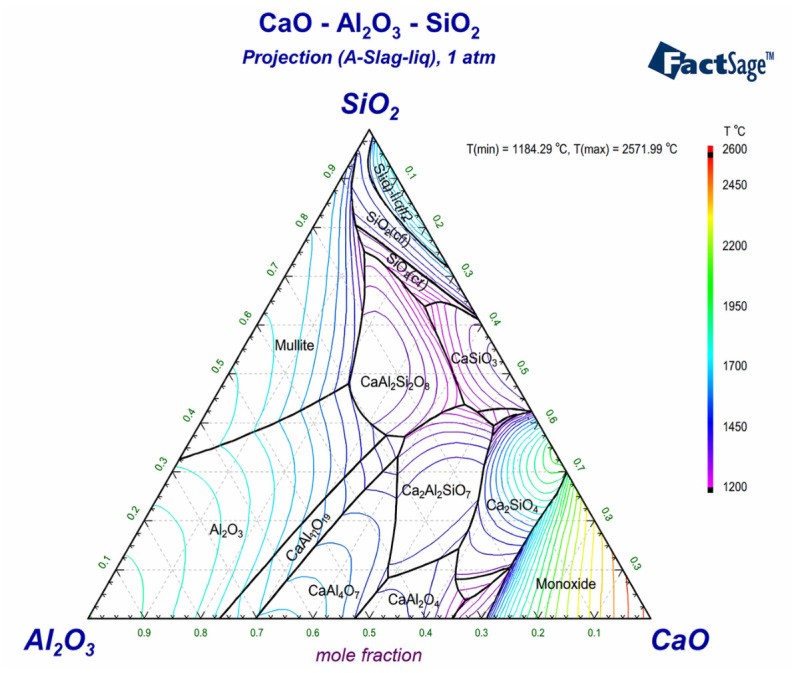
CaO-SiO_2_-Al_2_O_3_ Ternary Phase Diagram.

**Figure 2:**
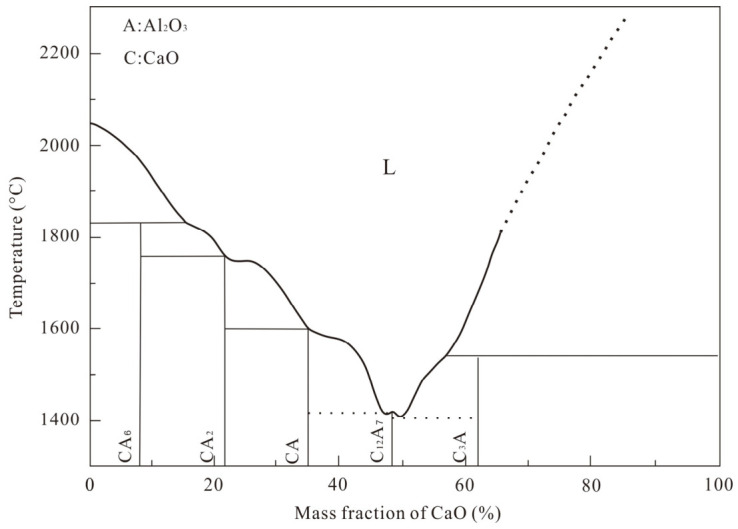
CaO-Al_2_O_3_ Binary Phase Diagram.

To mitigate severe slag–steel interfacial reactions during the continuous casting of rare-earth weathering steels, reducing the intrinsic reactivity of the mold flux is a critical design objective. Thermodynamic analysis indicates that the standard Gibbs free energy for the reaction between rare-earth Ce and SiO_2_ is significantly lower than that for its reactions with CaO and Al_2_O_3_, implying a much stronger reaction tendency between rare-earth elements and SiO_2_ [11]. Consequently, in the present work, the composition design strategy was shifted from the conventional CaO-SiO_2_-based low-melting region to the CaO-Al_2_O_3_-based low-melting region (Figure 1). According to the CaO-Al_2_O_3_ phase diagram in Figure 2, and considering both melting behavior and compositional tunability, the baseline composition was defined by a *w*(CaO)/*w*(Al_2_O_3_) ratio in the range of 0.6–2.0.

**Figure 3:**
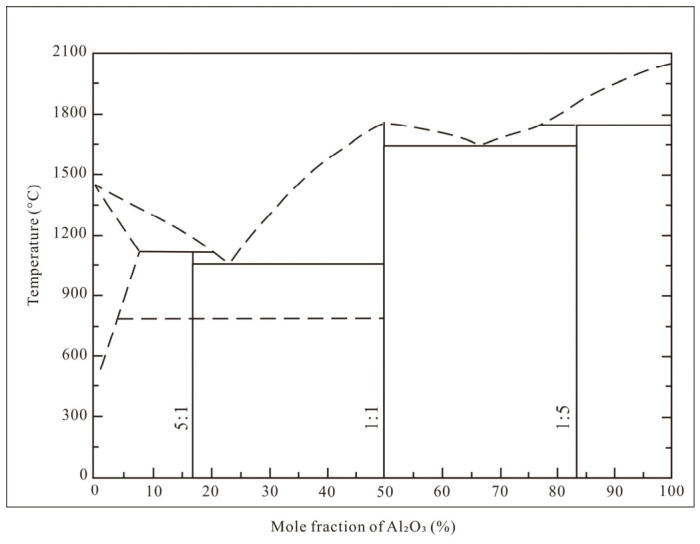
Li_2_O–Al_2_O_3_ Binary phase diagram.

However, the CaO-Al_2_O_3_ base system inherently exhibits a relatively high melting temperature, which necessitates the introduction of suitable fluxing agents to reduce the melting temperature and to adjust other physicochemical properties. Thermodynamic evaluation shows that Na_2_O has a strong reaction tendency with rare-earth Ce, as indicated by its large negative Gibbs free energy of reaction, and was therefore excluded from the flux design. In contrast, Li_2_O exhibits a much weaker interaction with rare-earth Ce and can effectively suppress the reaction between the mold flux and rare-earth elements while maintaining the stability of slag properties. Furthermore, the Li_2_O-Al_2_O_3_ phase diagram indicates that Li_2_O can form low-melting-point compounds with Al_2_O_3_ in Figure 3, thereby significantly reducing the melting temperature of the slag [12,13,14]. Based on these considerations, Li_2_O was selected as the primary fluxing agent, and its content was controlled within the range of 5–15 wt.%.

**Figure 4:**
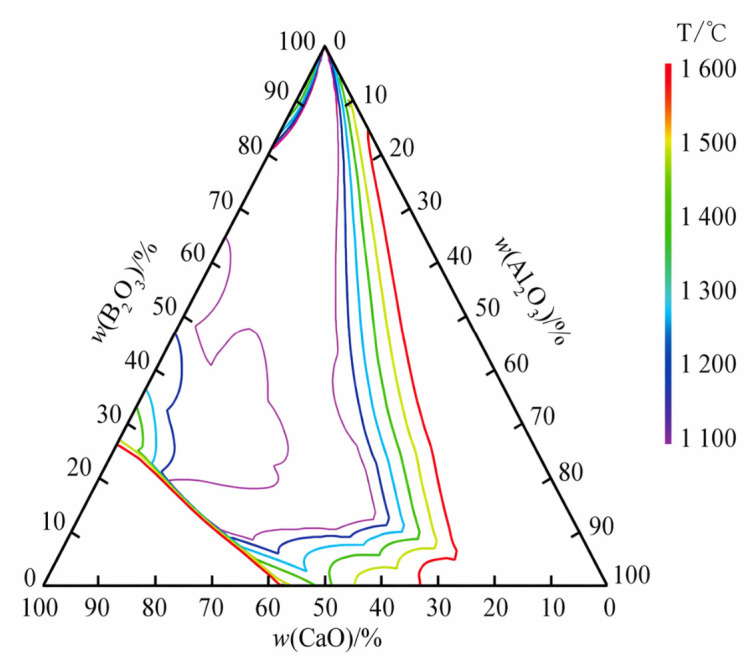
Isothermal Section of the CaO-Al_2_O_3_-B_2_O_3_ System.

In addition, B_2_O_3_, which has a low melting point of approximately 450 °C and readily forms low-melting compounds with various oxides, was introduced to further decrease the melting temperature of the slag system [15,16,17]. Phase diagram analysis of the CaO-Al_2_O_3_-B_2_O_3_ system shows that increasing the B_2_O_3_ content from 0 to 10 wt.% can reduce the melting temperature of the system by as much as ~500 °C in Figure 4. Therefore, considering both melting performance and compositional stability, the B_2_O_3_ content in the new mold flux was determined to be in the range of 0–10 wt.%.

To further suppress the reaction between rare-earth elements and the mold flux, in addition to lowering the activity of the reactants, the reaction driving force can also be reduced by increasing the activity of the reaction products [18,19]. On this basis, an appropriate amount of rare-earth oxide Ce_2_O_3_ was introduced into the mold flux to inhibit further transfer and reaction of rare-earth elements into the slag and to reduce the overall reactivity of the slag system. Taking into account both thermodynamic considerations and process adaptability, the Ce_2_O_3_ content was controlled within the range of 0–20 wt.%.

On the basis of the above design principles, a novel low-reactivity mold flux system with the composition CaO-Al_2_O_3_-Li_2_O-B_2_O_3_-Ce_2_O_3_ was finally established.

## 3. Thermodynamic Analysis of Slag Components

### 3.1. Model Assumptions

Based on the Ion and Molecule Coexistence Theory (IMCT), the CaO-Al_2_O_3_-Li_2_O-B_2_O_3_-Ce_2_O_3_ slag system was analyzed by integrating published thermodynamic information with calculations performed using FactSage 8.2, and the corresponding structural units of the melt were identified as summarized in Table 1 [20,21]. After determining the structural units that may exist in the molten slag, equilibrium-constant expressions were formulated for all relevant reactions, and an activity-calculation model for the mold flux was subsequently established according to the principle of mass balance.

Assume that $m_{1}=\sum X_{CaO},m_{2}=\sum X_{Al_{2}O_{3}},m_{3}=\sum X_{Li_{2}O},m_{4}=\sum X_{B_{2}O_{3}},\;m_{5}=\sum X_{Ce_{2}O_{3}},N_{1}=N_{CaO},N_{2}=N_{Al_{2}O_{3}},N_{3}=N_{Li_{2}O},N_{4}=N_{B_{2}O_{3}},N_{5}=N_{C_{2}O_{3}},\;N_{6}=N_{CaO6Al_{2}O_{3}},N_{7}=N_{CaOAl_{2}O_{3}},N_{8}=N_{3CaOAl_{2}O_{3}},N_{9}=N_{12CaO7Al_{2}O_{3}},N_{10}=N_{CaO2Al_{2}O_{3}},\;N_{11}=N_{2Al_{2}O_{3}\cdot B_{2}O_{3}},N_{12}=N_{9Al_{2}O_{3}\cdot 2B_{2}O_{3}},N_{13}=N_{CaO2B_{2}O_{3}},N_{14}=N_{CaOB_{2}O_{3}},N_{15}=N_{2CaOB_{2}O_{3}},\;N_{16}=N_{3CaOB_{2}O_{3}},N_{17}=N_{Ca_{2}O_{3}\cdot Al_{2}O_{3}},N_{18}=N_{C_{2}O_{3}\cdot 11H_{2}O_{3}},N_{19}=N_{Li_{2}O\cdot dl_{2}O_{3}},N_{20}=N_{Li_{2}O\cdot B_{2}O_{3}},\;N_{21}=N_{Li_{2}O\cdot 2B_{2}O_{3}},N_{22}=N_{Li_{2}O\cdot 3B_{2}O_{3}},N_{23}=N_{Li_{2}O\cdot 4B_{2}O_{3}}.$

Here, ∑X denotes the total moles of all structural units at equilibrium for an assumed 100 g of slag. xi (i = 1, 2, 3… 22) represents the moles of a given substance in the melt after reaction equilibrium is reached. mi (i = 1, 2, 3, 4, 5) denotes the total moles of CaO, Al_2_O_3_, Li_2_O, B_2_O_3_, and Ce_2_O_3_, respectively, before reaction. Ni (i = 1, 2, 3, 4, 5) is the effective concentration of each component in the slag, which is defined as the activity of that component.

According to the Ion and Molecule Coexistence Theory, CaO, Ce_2_O_3_, and Li_2_O exist as face-centered cubic ionic lattices in the solid state, and in the molten slag these compounds occur as ionic species such as Ca^2+^, Ce^3+^, Li^+^, and O^2−^, remaining independent rather than existing in molecular form.

The mass-action concentration expressions of the structural units and the corresponding equilibrium reactions used in the model are summarized in Table 2.

According to the mass-balance principle:

(24) $$\sum_{i=1}^{23}\;N_{i}=1$$

Each component obeys the law of mass conservation, and because the total moles of CaO remain unchanged before and after reaction, Equation (25) can be obtained.

(25) $$\sum X=\frac{m_{1}}{N_{1}/2+N_{6}+N_{7}+3N_{8}+12N_{9}+N_{10}+N_{13}+N_{14}+2N_{15}+3N_{16}}$$

Because the total moles of Al_2_O_3_ remain unchanged before and after reaction, Equation (26) can be obtained.

(26) $$\sum X=\frac{m_{2}}{N_{2}+6N_{6}+N_{7}+N_{8}+7N_{9}+2N_{10}+9N_{11}+N_{17}+11N_{18}+N_{19}}$$

Because the total moles of Li_2_O remain unchanged before and after reaction, Equation (27) can be obtained.

(27) $$\sum X=\frac{m_{3}}{N_{3}/3+N_{19}+N_{20}+N_{21}+N_{22}}$$

Because the total moles of B_2_O_3_ remain unchanged before and after reaction, Equation (28) can be obtained.

(28) $$\sum X=\frac{m_{4}}{N_{4}+N_{11}+2N_{12}+2N_{13}+N_{14}+N_{15}+N_{16}+N_{20}+2N_{21}+3N_{22}}$$

Because the total moles of Ce_2_O_3_ remain unchanged before and after reaction, Equation (29) can be obtained.

(29) $$\sum X=\frac{m_{5}}{N_{5}/5+N_{17}+N_{18}}$$

Equations (1)–(29) (Table 2) constitute the activity-calculation model for the CaO–Al_2_O_3_–Li_2_O–B_2_O_3_–Ce_2_O_3_ system. Finally, the effective concentrations of all structural units can be calculated using the fsolve function in Matlab 2024.

The nonlinear system of equations was solved using Matlab, and the Newton iterative method was adopted as the computational approach. The Newton iterative method is a successive linearization technique for solving the nonlinear system f(x) = 0. Performing a Taylor expansion at an approximate solution x^(k) yields

(30) $$f\left(x\right)\approx f\left(x^{\left(k\right)}\right)+f^{\prime }\left(x^{\left(k\right)}\right)\left(x-x^{\left(k\right)}\right)$$

Thus, the approximate equation for f(x) = 0 can be expressed as

(31) $$f\left(x\right)\approx f\left(x^{\left(k\right)}\right)+f^{\prime }\left(x^{\left(k\right)}\right)\left(x-x^{\left(k\right)}\right)$$

where f′(x^(k)) is the Jacobian matrix of f(x), given by

(32) $$f^{\prime }\left(x^{\left(k\right)}\right)={\left[\begin{array}{cccc} \frac{\partial f_{1}}{\partial x_{1}} & \frac{\partial f_{1}}{\partial x_{2}} & \Lambda & \frac{\partial f_{1}}{\partial x_{n}}\\ \frac{\partial f_{2}}{\partial x_{1}} & \frac{\partial f_{2}}{\partial x_{2}} & \Lambda & \frac{\partial f_{2}}{\partial x_{n}}\\ \vdots & \vdots & \ddots & \vdots \\ \frac{\partial f_{n}}{\partial x_{1}} & \frac{\partial f_{n}}{\partial x_{2}} & \Lambda & \frac{\partial f_{n}}{\partial x_{n}} \end{array}\right]}_{x=x^{\left(k\right)}}$$

If the Jacobian matrix is nonsingular, the unique solution of the corresponding linearized system can be written as

(33) $$x^{\left(k+1\right)}=x^{\left(k\right)}-\lambda {\left[f^{\prime }\left(x^{\left(k\right)}\right)\right]}^{-1}f\left(x^{\left(k\right)}\right)\;\left(k=0,1,2,\Lambda \right)$$

where Equation (33) represents the Newton iterative scheme for solving the nonlinear system f(x) = 0.

### 3.2. Model Validation

**Figure 5:**
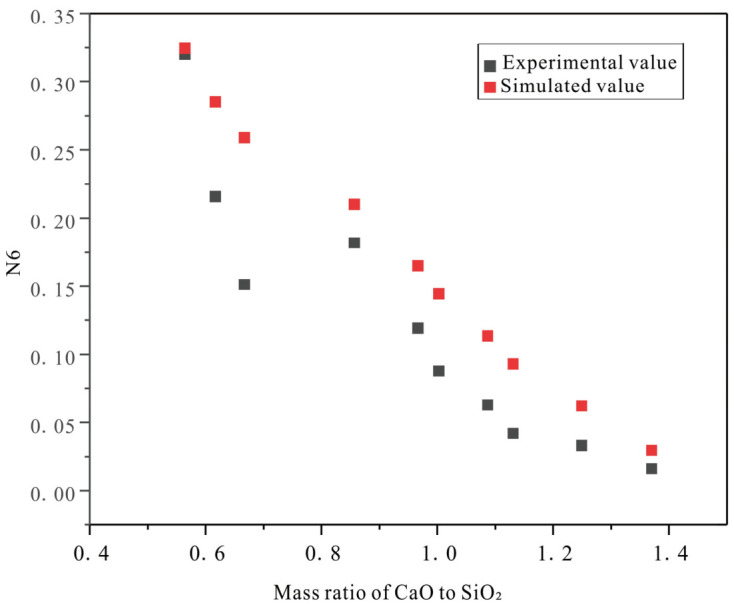
Comparison between measured value and calculated value of activity of SiO_2_.

**Figure 6:**
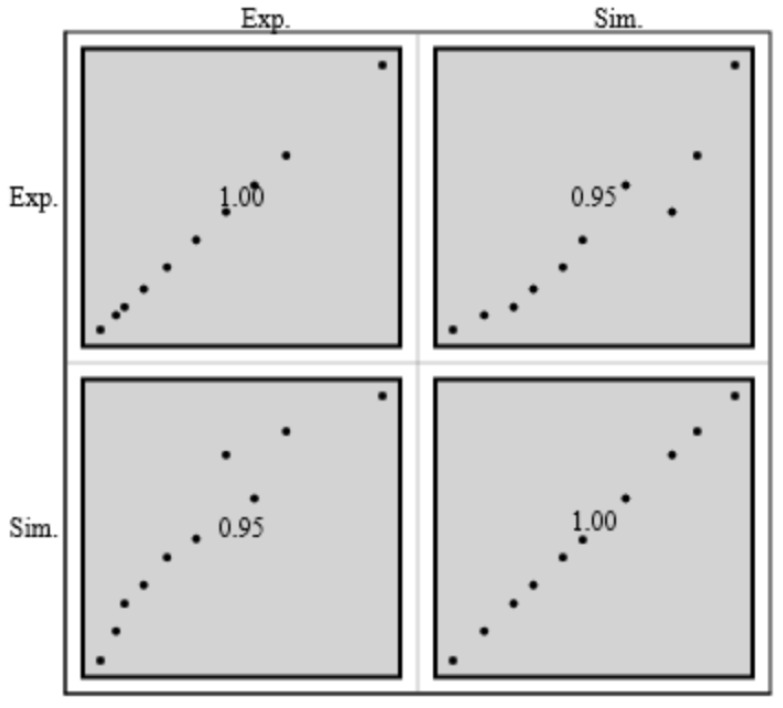
Pearson correlation analysis between the experimentally measured and model-calculated SiO_2_ activities.

Owing to the complexity of the slag-system compositions calculated by the present model, direct determination of the activities of its constituent components is difficult; therefore, in this study, the experimentally measured SiO_2_ activity values for the Al_2_O_3_–CaF_2_–CaO–SiO_2_ slag system at 1823 K reported in Ref. [22] were compared with the values calculated by the present model, and a Pearson correlation analysis was performed. As shown in Figure 5 and Figure 6, Pearson correlation analysis of the experimentally measured values and the model-calculated values indicates that the two vary in the same direction, that is, when the experimental values increase, the simulated values also increase correspondingly; the correlation coefficient is 0.95, indicating an extremely strong positive correlation, as |r| ≥ 0.8 is generally considered to represent a strong correlation. This result demonstrates excellent consistency between the experimental observations and the simulation predictions, indicating that the simulation model can reliably reflect the experimental behavior.

### 3.3. Activities of Slag Components Under Different Conditions

**Figure 7:**
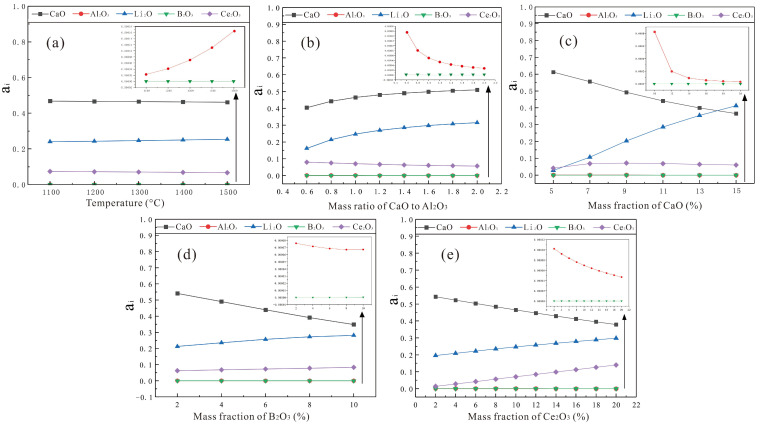
Activities of individual components in the slag system under different conditions: (**a**) temperature; (**b**) *w*(CaO)/*w*(Al_2_O_3_); (**c**) *w*(Li_2_O); (**d**) *w*(B_2_O_3_); (**e**) *w*(Ce_2_O_3_).

Based on the established computational framework, the baseline mold flux with the composition listed in Table 3 was selected as the reference system. The activities of the primary oxide components in the newly designed flux (CaO, Al_2_O_3_, Li_2_O, B_2_O_3_, and Ce_2_O_3_), together with those of the major composite oxides potentially present in the slag (e.g., Li_2_O·Al_2_O_3_, 3CaO·B_2_O_3_, and 2CaO·B_2_O_3_), were calculated under varying temperatures and compositional conditions. The results are summarized in Figure 7.

#### 3.3.1. Effect of Temperature on Component Activities

The calculations indicate that CaO, Li_2_O, and Ce_2_O_3_ exhibit relatively high activities in the novel mold flux, whereas Al_2_O_3_ shows low activity and B_2_O_3_ remains close to zero across the investigated temperature range. The slag is predicted to contain several stable composite oxides, including Li_2_O·Al_2_O_3_, 3CaO·B_2_O_3_, and 2CaO·B_2_O_3_. The low reactivity of the mold flux can be rationalized from two aspects. First, low-reactivity constituents such as CaO and Li_2_O retain high activities, while the comparatively more reactive species B_2_O_3_ is thermodynamically suppressed to an extremely low activity, thereby reducing the overall driving force for slag reactions. Second, Ce_2_O_3_ is a key product of slag–metal interfacial reactions; its relatively high activity is expected to impede further interfacial reaction progress.

Notably, the activities of Al_2_O_3_ and B_2_O_3_ remain extremely low at all temperatures. This behavior is primarily attributed to the relatively high CaO fraction in the flux, which promotes the association of Al_2_O_3_ and B_2_O_3_ with CaO to form stable composite oxides. Consequently, the equilibrium concentrations of Al_2_O_3_ and B_2_O_3_ in the melt are substantially reduced, driving their activities toward zero.

#### 3.3.2. Effect of *w*(CaO)/*w*(Al_2_O_3_) on Component Activities

Figure 7b shows the activity evolution of flux components as a function of *w*(CaO)/*w*(Al_2_O_3_). With increasing *w*(CaO)/*w*(Al_2_O_3_), the activities of CaO and Li_2_O increase markedly, Ce_2_O_3_ decreases slightly, Al_2_O_3_ remains at an extremely low level and continues to decrease, and B_2_O_3_ stays near zero. These trends arise mainly from the compositional shift induced by increasing *w*(CaO)/*w*(Al_2_O_3_): the relative mass fraction of CaO increases while that of Al_2_O_3_ decreases, leading to a higher activity of CaO and a lower activity of Al_2_O_3_. In parallel, the reduced Al_2_O_3_ availability lowers the equilibrium concentrations of Al_2_O_3_-bearing composite oxides such as Li_2_O·Al_2_O_3_ and CaO·Al_2_O_3_, resulting in decreased activities of these compounds. Moreover, because less Li_2_O is consumed to form Li_2_O·Al_2_O_3_, a larger fraction of free Li_2_O remains in the melt, thereby increasing its activity. Overall, within an appropriate compositional window, elevating *w*(CaO)/*w*(Al_2_O_3_) increases the activities of the low-reactivity constituents CaO and Li_2_O and thus contributes to reducing flux reactivity.

#### 3.3.3. Effect of Fluxing-Agent Content (Li_2_O and B_2_O_3_) on Component Activities

Figure 7c presents the activities of flux constituents at different Li_2_O mass fractions. Increasing Li_2_O leads to a pronounced rise in Li_2_O activity, while Ce_2_O_3_ remains nearly constant and CaO and Al_2_O_3_ decrease slightly; B_2_O_3_ continues to exhibit an activity close to zero. The elevated Li_2_O activity enhances its interaction with Al_2_O_3_, promoting the formation of Li_2_O·Al_2_O_3_. As a result, the activity of Li_2_O·Al_2_O_3_ increases, accompanied by a further decrease in Al_2_O_3_ activity. With continued Li_2_O addition, the higher equilibrium concentration of Li_2_O·Al_2_O_3_ suppresses the formation of other Al_2_O_3_-containing composite oxides (e.g., CaO·Al_2_O_3_, 3CaO·Al_2_O_3_, and Ce_2_O_3_·Al_2_O_3_), leading to reduced equilibrium concentrations and activities of these phases.

Figure 7d shows the activity changes induced by varying B_2_O_3_ content. As B_2_O_3_ increases, the activities of Li_2_O and Ce_2_O_3_ increase (the former more significantly), whereas CaO and Al_2_O_3_ decrease. When B_2_O_3_ is below ~12 wt.%, its activity remains nearly zero, which is attributed to the strong stabilization of B_2_O_3_ through the formation of calcium borates (2CaO·B_2_O_3_ and 3CaO·B_2_O_3_). Above ~12 wt.% B_2_O_3_, the activity of B_2_O_3_ shows a slight increase but still remains at a very low level overall. Meanwhile, increasing B_2_O_3_ shifts the dominant borate species from 3CaO·B_2_O_3_ toward 2CaO·B_2_O_3_, as reflected by the increasing activity of 2CaO·B_2_O_3_ and the decreasing activity of 3CaO·B_2_O_3_.

#### 3.3.4. Effect of Rare-Earth Oxide (Ce_2_O_3_) on Component Activities

Figure 7e illustrates the effect of Ce_2_O_3_ mass fraction on component activities. Increasing Ce_2_O_3_ markedly increases the activities of Ce_2_O_3_ and Li_2_O, while decreasing those of CaO and Al_2_O_3_; B_2_O_3_ remains close to zero. The increase in Ce_2_O_3_ activity is a direct consequence of its higher concentration. In addition, Ce_2_O_3_ tends to associate with Al_2_O_3_ to form Ce_2_O_3_·Al_2_O_3_, which lowers Al_2_O_3_ activity while increasing the activity of Ce_2_O_3_·Al_2_O_3_. As Ce_2_O_3_·Al_2_O_3_ formation intensifies, the formation of Li_2_O·Al_2_O_3_ is comparatively reduced, causing a decrease in Li_2_O·Al_2_O_3_ activity and a concomitant increase in Li_2_O activity. Moreover, the slight decrease in CaO activity with increasing Ce_2_O_3_ promotes the conversion of 3CaO·B_2_O_3_ to 2CaO·B_2_O_3_. Collectively, increasing Ce_2_O_3_ raises the activities of the low-reactivity constituents Li_2_O and Ce_2_O_3_, providing a thermodynamic basis for mitigating slag–metal interfacial reactions and reducing the overall reactivity of the mold flux.

## 4. Composition Optimization of the Slag System and Investigation of Its Physicochemical Properties

### 4.1. Methodology

#### 4.1.1. Preparation of Slag Samples

In this chapter, mold fluxes with different compositions were prepared by premelting reagent-grade pure chemicals. To remove moisture and impurities from the reagents, each reagent was calcined in a muffle furnace (National Engineering Research Center for Continuous Casting Technology, Beijing, China) at high temperature for 2 h. The calcined chemical reagents were weighed according to the designed compositions, thoroughly mixed, placed into a graphite crucible, and heated in a high-temperature tube furnace(National Engineering Research Center for Continuous Casting Technology, Beijing, China) at 1200 °C to 1450 °C, where they were held for 1 h to ensure complete melting of the slag samples. After premelting, the mold fluxes were quenched, dried, ground, and sieved to obtain a particle size of less than 0.074 mm for subsequent physicochemical property measurements.

#### 4.1.2. Method for Viscosity Measurement

**Figure 8:**
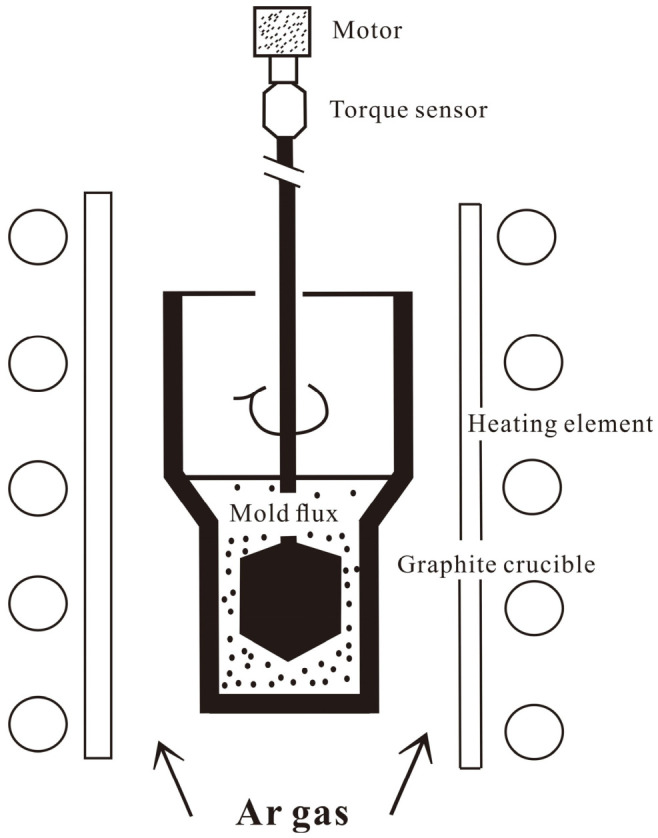
Apparatus sketch for measuring the viscosity of mold flux.

The viscosity characteristics of the mold fluxes were measured using the rotating cylinder method, and the experimental apparatus was an RTW-10 comprehensive melt property tester (National Engineering Research Center for Continuous Casting Technology, Beijing, China), as shown in Figure 8. The main heating unit was a MoSi_2_ high-temperature furnace, and an S-type thermocouple was used for furnace temperature control and measurement, with a temperature control accuracy of ±0.5 °C. The temperature-measuring thermocouple was positioned at the bottom of the graphite crucible. The graphite crucible used to contain the slag had an outer diameter of 50 mm, an inner diameter of 40 mm, and a height of 80 mm.

The viscosity measurement procedure was as follows.

(1) Before heating, the graphite crucible lined with a molybdenum sheet was placed in the constant-temperature zone of the furnace, and approximately 140 g of premelted slag was weighed and loaded into the crucible. The power supply and control program were then turned on, and the temperature was raised to 1350 °C and held for 30 min to ensure complete melting of the slag sample.

(2) A molybdenum rod was used to stir the molten slag and adjust the slag height to 40 mm, after which the Mo probe and suspension rod were installed so that they were positioned exactly at the center of the furnace tube. The furnace height was then adjusted so that the distance between the lowest end of the Mo probe and the bottom of the graphite crucible was 10 mm.

(3) The viscosity measurement system was started, and the Mo probe was rotated at 200 rpm while the furnace temperature was decreased at a rate of 3 °C/min for viscosity measurement and data recording. The measurement was terminated when the viscosity increased to 10 Pa·s.

(4) After completion of the viscosity measurement, the furnace temperature was raised to a level at which the slag viscosity was relatively low, the molybdenum probe was removed, and the experiment was concluded. To protect the graphite crucible and sleeve, argon was used throughout the experiment as a protective atmosphere.

At high temperatures, the relationship between the viscosity of the mold flux and temperature follows the Arrhenius equation, showing a linear functional relationship. However, when the structure of the mold flux changes, such as when crystalline phases precipitate, the relationship between viscosity and temperature no longer follows the Arrhenius equation. Therefore, the temperature at which the functional relationship between viscosity and temperature begins to deviate from linearity is defined as the break temperature of the continuous casting mold flux. In addition, the apparent activation energy for viscous flow of the mold flux can be obtained by fitting the relationship between viscosity and temperature in the high-temperature region. The activation energy for viscous flow represents the energy required for one mole of particles in the molten slag to move from one equilibrium position to another.

#### 4.1.3. Method for Testing Crystalline Phases

The raw material used in the crystalline phase test was premelted slag. An appropriate amount of slag sample was taken and fully melted at 1350 °C. Subsequently, with reference to the viscosity–temperature curve obtained during continuous cooling, the fully molten slag sample was cooled at a rate of 3 °C/min, and samples were collected at different characteristic temperatures according to the specific scheme. At the break temperature, slag samples were taken and water-quenched to analyze the initial crystalline phases of the mold flux. At the temperature corresponding to a viscosity of 10 Pa·s, slag samples were taken and water-quenched, and phase analysis was conducted on the mold flux after complete crystallization. The phase identification and analysis techniques were as follows.

(1) X-ray diffraction (XRD) analysis.

Phase identification of the mold flux was carried out using a D/max-2500PC X-ray diffractometer (Rigaku Corporation, Akishima, Tokyo, Japan) under the following conditions: Cu target Kα radiation with a wavelength of λ = 1.544426 Å, an operating voltage of 40 kV, a 2θ scanning range of 10–90°, and a scanning rate of 0.033°·s^−1^.

#### 4.1.4. Method for Measuring Melting Temperature

The melting temperature of the mold flux was determined by the hemisphere point method. As the temperature increased, the amount of liquid phase in the slag gradually increased and the sample softened, causing its shape to change progressively and its height to decrease continuously. After complete melting, the sample spread over the substrate. the temperature at which the sample height decreases to one-half of its original height is defined as the melting temperature.

**Figure 9:**
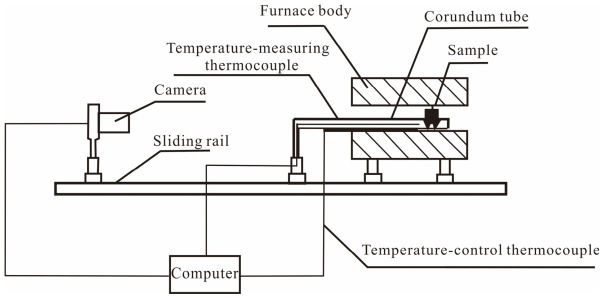
Apparatus sketch for measuring the melting temperature of mold flux.

The melting temperature of the mold flux was measured using a melting point and melting rate analyzer (National Engineering Research Center for Continuous Casting Technology, Beijing, China), and the experimental apparatus is shown in Figure 9. The equipment mainly consisted of a heating system, a temperature control system, an imaging system, and a rail transmission system. The heating device was a horizontal tube furnace equipped with U-shaped MoSi_2_ heating elements, with a maximum operating temperature of 1550 °C. The furnace temperature was controlled by silicon-controlled rectifier components, with a temperature control accuracy of ±2 °C. The optical imaging system was an OLYMPUS high-definition camera. The transmission system was used to move the sample into and out of the heating furnace.

The melting temperature measurement procedure was as follows.

(1) A small amount of premelted slag with a particle size of less than 0.074 mm was mixed with alcohol and pressed into a cylindrical specimen with both diameter and height of 3 mm. The specimen was then placed at the center of an alumina substrate, and the alumina substrate was positioned in the groove of an alumina tube so that the temperature-measuring thermocouple was located directly beneath the alumina substrate.

(2) The temperature control program was set and operated so that the furnace heating rate was 10 °C/min. When the furnace temperature reached 550 °C, the slag sample was introduced into the constant-temperature zone of the furnace, and the positions of the eyepiece and objective lens were adjusted so that a clear projected image appeared on the computer screen, after which the image coordinates were entered.

(3) The temperature corresponding to the point at which the specimen height decreased to one-half of its original height during heating was recorded.

(4) The specimen was removed, and the temperature control program was turned off so that the furnace temperature decreased to room temperature.

### 4.2. Effect of w(CaO)/w(Al_2_O_3_) on Physicochemical Properties of the Mold Flux

**Figure 10:**
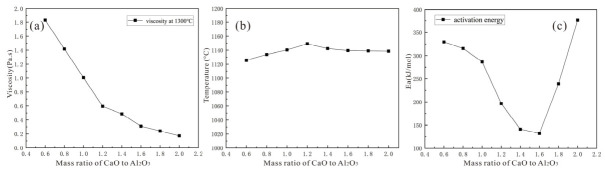
Performance parameters of the mold flux under different *w*(CaO)/*w*(Al_2_O_3_) ratios: (**a**) viscosity; (**b**) temperature; (**c**) activation energy.

Guided by the theoretical calculations, the *w*(CaO)/*w*(Al_2_O_3_) ratio of the slag system was systematically adjusted to improve the physicochemical performance of the mold flux. The corresponding melting temperatures are presented in Figure 10a. As *w*(CaO)/*w*(Al_2_O_3_) increased, the melting temperature exhibited a non-monotonic evolution, increasing initially and then decreasing before approaching a quasi-stable value. The highest melting temperature (1149.2 °C) was obtained at *w*(CaO)/*w*(Al_2_O_3_) = 1.2, whereas the lowest value (1125.5 °C) occurred at *w*(CaO)/*w*(Al_2_O_3_) = 0.6. When *w*(CaO)/*w*(Al_2_O_3_) was within 1.4–2.0, the melting temperature remained nearly constant at approximately 1140 °C.

**Figure 11:**
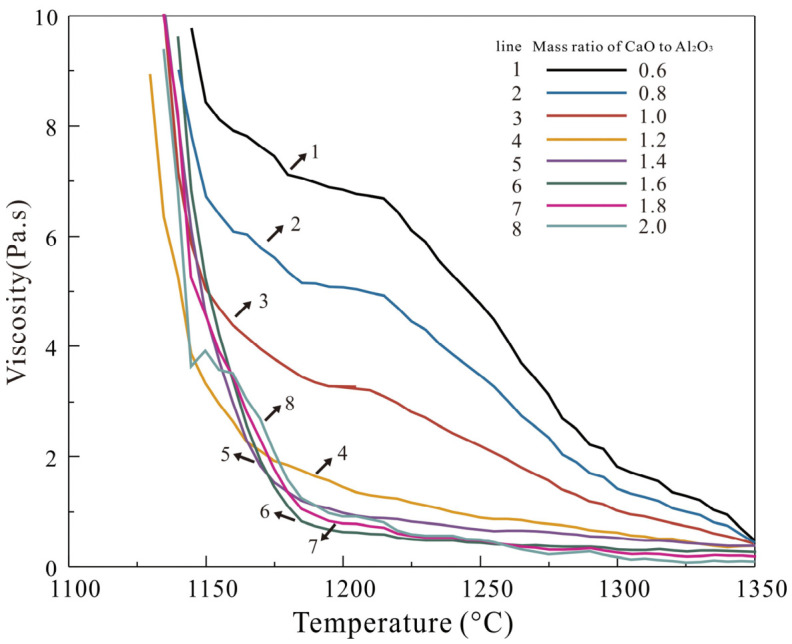
Viscosity temperature curve of mold flux with different *w*(CaO)/*w*(Al_2_O_3_).

**Figure 12:**
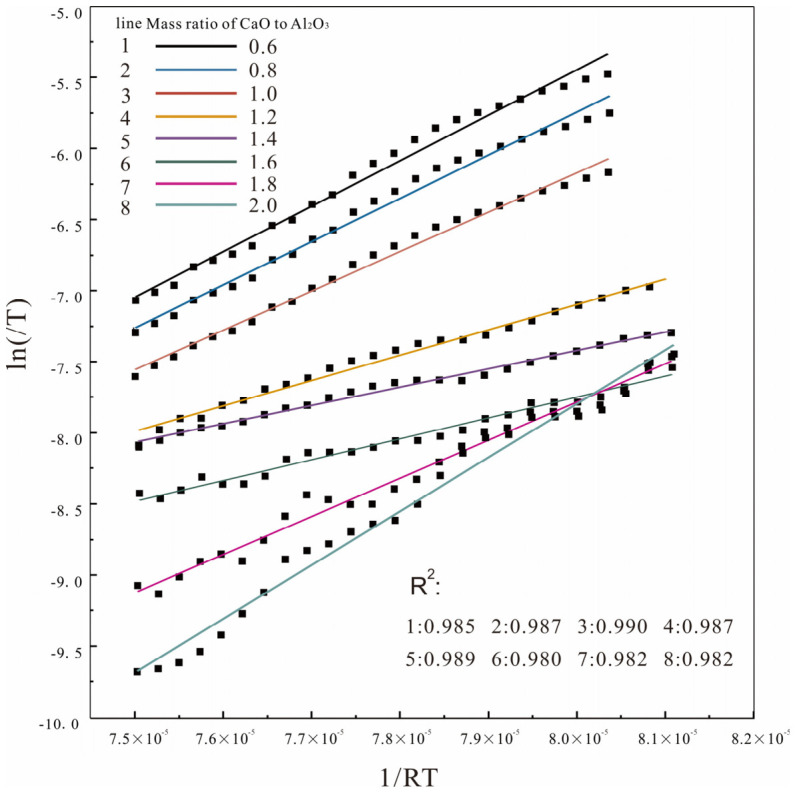
Functional relationship between mold flux viscosity and temperature under different *w*(CaO)/*w*(Al_2_O_3_) ratios.

Figure 10 shows the performance parameters of the mold flux under different *w*(CaO)/*w*(Al_2_O_3_) ratios. Figure 11 presents the viscosity–temperature curves of the mold flux at different *w*(CaO)/*w*(Al_2_O_3_) ratios. Figure 12 shows the fitting results for the relationship between mold flux viscosity and temperature. The viscosity–temperature behavior was strongly dependent on *w*(CaO)/*w*(Al_2_O_3_). For *w*(CaO)/*w*(Al_2_O_3_) = 0.6–1.0, viscosity decreased markedly with increasing temperature below 1300 °C, and the viscosity at 1300 °C exceeded 1 Pa·s; no distinct inflection point was observed in the viscosity curve. At *w*(CaO)/*w*(Al_2_O_3_) = 1.2, viscosity decreased rapidly with temperature up to ~1200 °C and then decreased more gradually at higher temperatures, again without a clear inflection. In contrast, when *w*(CaO)/*w*(Al_2_O_3_) increased to 1.4–2.0, a pronounced inflection emerged at around 1200 °C, indicating a transition in flow behavior; prior to the inflection point, viscosity decreased sharply with increasing temperature, whereas beyond the inflection it became relatively stable. Among these compositions, the flux at *w*(CaO)/*w*(Al_2_O_3_) = 1.60 showed the most stable viscosity before the inflection point, suggesting improved operational stability.

At 1300 °C, the viscosity decreased with increasing *w*(CaO)/*w*(Al_2_O_3_) and then exhibited a slight rebound before remaining essentially constant; when *w*(CaO)/*w*(Al_2_O_3_) exceeded 1.6, the viscosity stayed at a low level of ~0.25 Pa·s. The apparent activation energy for viscous flow decreased first and then increased with increasing *w*(CaO)/*w*(Al_2_O_3_), reaching a minimum of 132.1 kJ·mol^−1^ at *w*(CaO)/*w*(Al_2_O_3_) = 1.6, which is consistent with enhanced melt fluidity in this compositional window.

Combined with the analysis of melt structure, as the *w*(CaO)/*w*(Al_2_O_3_) ratio increases, the mass fraction of CaO in the slag increases, the proportion of O^2−^ ions rises, and the degree of dissociation of the mold flux is enhanced. Moreover, the increase in O^2−^ ions promotes the formation of two-dimensional BO_3_^3−^ triangular structural units, disrupts the three-dimensional network structure, and leads to a decreasing trend in the viscosity of the mold flux.

### 4.3. Effect of Fluxing-Agent Content on Physicochemical Properties of the Mold Flux

**Figure 13:**
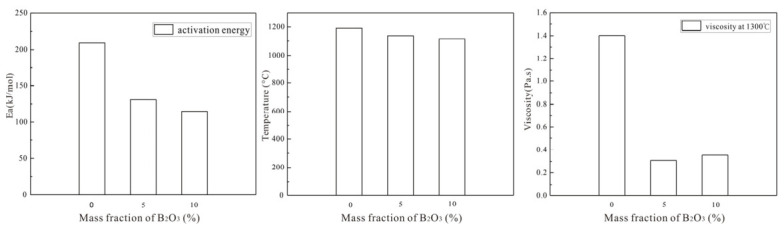
Performance parameters of the mold flux at different B_2_O_3_ mass fractions.

The influence of B_2_O_3_ content on melting temperature is shown in Figure 13. The melting temperature decreased progressively with increasing B_2_O_3_ mass fraction and reached a minimum of 1118 °C at 10 wt.% B_2_O_3_. Relative to the B_2_O_3_-free composition, the addition of 5 wt.% B_2_O_3_ reduced the melting temperature by 50.7 °C; increasing B_2_O_3_ from 5 to 10 wt.% produced an additional reduction of 22.4 °C, indicating that the fluxing efficiency of B_2_O_3_ diminishes at higher additions.

**Figure 14:**
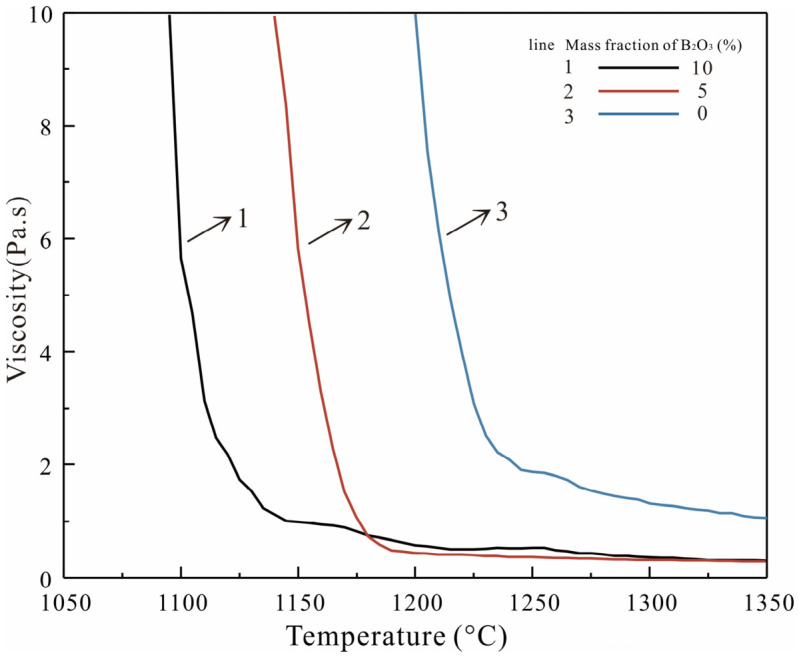
Viscosity–temperature curves of the mold flux at different B_2_O_3_ mass fractions.

**Figure 15:**
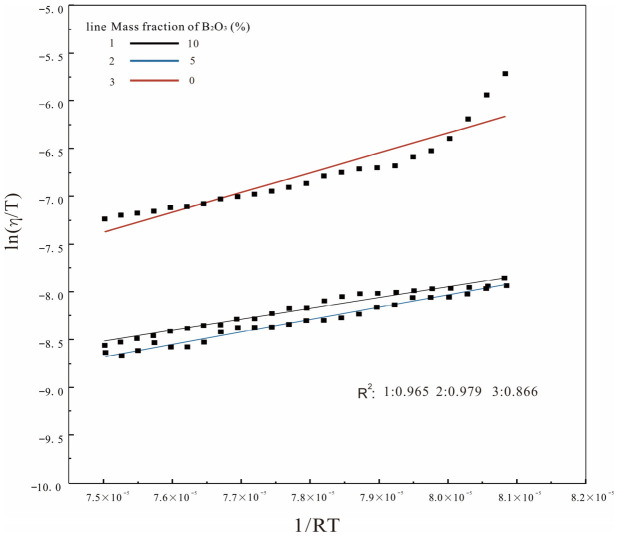
Functional relationship between mold flux viscosity and temperature at different B_2_O_3_ mass fractions.

Figure 13 shows the performance parameters of the mold flux at different mass fractions of B_2_O_3_. Figure 14 presents the viscosity–temperature curves of the mold flux under different B_2_O_3_ mass fractions. Figure 15 shows the fitting results for the relationship between mold flux viscosity and temperature.

For all B_2_O_3_ levels, viscosity decreased with increasing temperature, while the inflection temperature shifted to lower values as B_2_O_3_ increased, reaching the lowest inflection temperature of 1145 °C at 10 wt.% B_2_O_3_. At 1300 °C, viscosity decreased and then slightly increased to an approximately constant value; when B_2_O_3_ exceeded 5 wt.%, viscosity remained low at ~0.33 Pa·s. The apparent activation energy for viscous flow decreased with increasing B_2_O_3_, reaching a minimum of 115.2 kJ·mol^−1^ at 10 wt.% B_2_O_3_, and remained essentially unchanged in the range of 5–10 wt.% B_2_O_3_.

Combined with the analysis of melt structure, it can be concluded that, in the absence of B_2_O_3_ addition, the slag contains a relatively large number of highly polymerized AlO_4_^5−^ tetrahedra, resulting in a high degree of polymerization of the mold flux as well as relatively high viscosity and viscous flow activation energy. When 5% B_2_O_3_ is added, the proportion of two-dimensional BO_3_^3−^ triangular structures increases, the degree of polymerization of the mold flux decreases significantly, and the viscosity is reduced. When the mass fraction of B_2_O_3_ increases from 5% to 10%, the viscosity increases slightly but remains relatively stable. When the mass fraction of B_2_O_3_ increases from 0 to 10%, the break temperature of the mold flux decreases continuously, mainly because B_2_O_3_ has an extremely low melting point, which can substantially reduce the melting temperature and increase the superheat of the molten slag.

**Figure 16:**
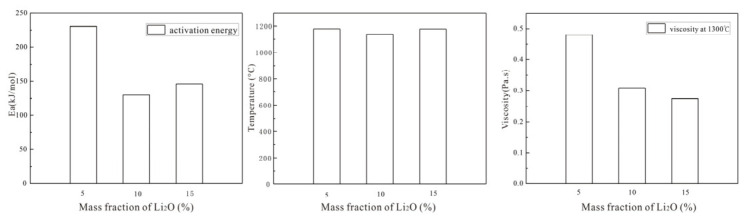
Performance parameters of the mold flux at different Li_2_O mass fractions.

The influence of Li_2_O content on melting temperature is shown in Figure 16. With increasing Li_2_O mass fraction, the melting temperature first decreased and then increased, reaching a minimum of 1139.5 °C at 10 wt.% Li_2_O.

**Figure 17:**
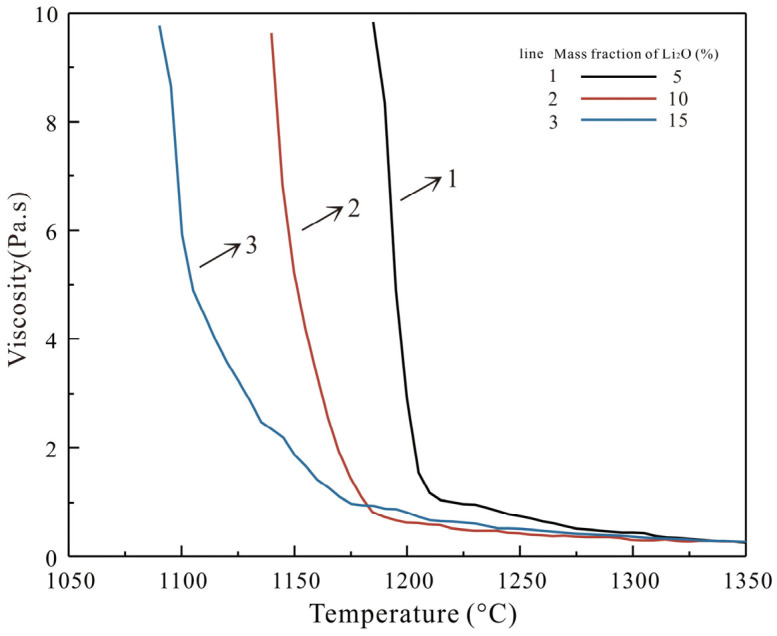
Viscosity–temperature curves of the mold flux at different Li_2_O mass fractions.

**Figure 18:**
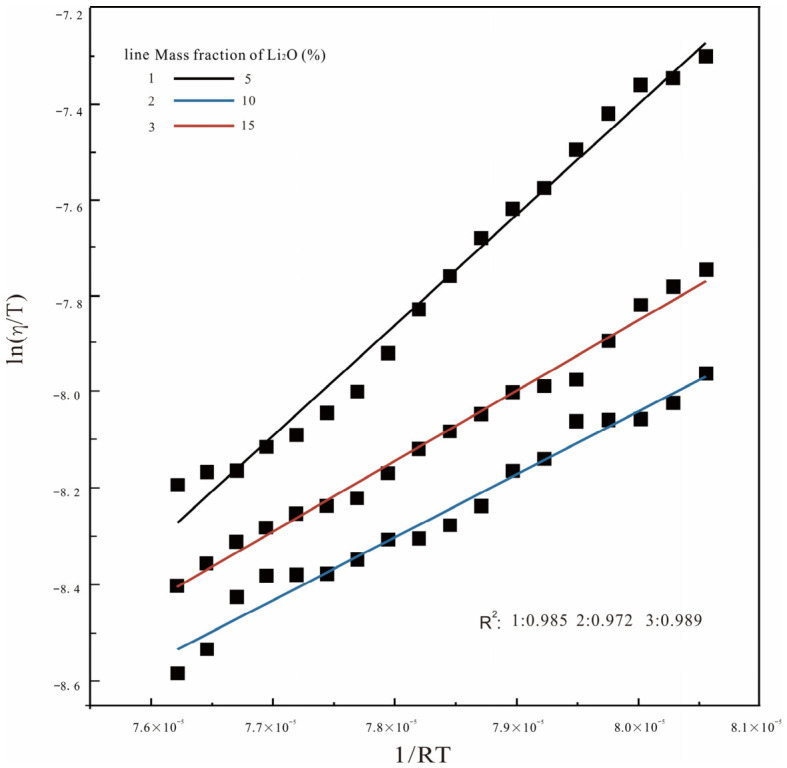
Functional relationship between mold flux viscosity and temperature at different Li_2_O mass fractions.

Figure 16 shows the performance parameters of the mold flux at different Li_2_O mass fractions. Figure 17 presents the viscosity–temperature curves of the mold flux under different Li_2_O mass fractions. Figure 18 shows the fitting results for the relationship between mold flux viscosity and temperature.

The viscosity results show that, for all Li_2_O levels, viscosity decreased with increasing temperature and the inflection temperature gradually decreased as Li_2_O increased, reaching a minimum of 1175 °C at 15 wt.% Li_2_O. When Li_2_O exceeded 10 wt.%, the inflection temperature became nearly invariant. The viscosity at 1300 °C decreased with increasing Li_2_O and reached a minimum of 0.2753 Pa·s at 15 wt.% Li_2_O; when Li_2_O was >10 wt.%, the viscosity remained at a low level of ~0.30 Pa·s. The apparent activation energy for viscous flow decreased first and then increased with increasing Li_2_O, reaching a minimum of 129.9 kJ·mol^−1^ at 10 wt.% Li_2_O and remaining essentially constant for 10–15 wt.% Li_2_O.

Combined with the analysis of melt structure, it can be concluded that, as the mass fraction of Li_2_O increases, the degree of polymerization of the mold flux shows a continuous decreasing trend, and consequently the viscosity of the mold flux continuously decreases. The break temperature gradually decreases, possibly because the increase in Li_2_O mass fraction weakens the crystallization ability of the mold flux and alleviates the abrupt change in its viscous characteristics.

### 4.4. Effect of Ce_2_O_3_ Content on Physicochemical Properties of the Mold Flux

**Figure 19:**
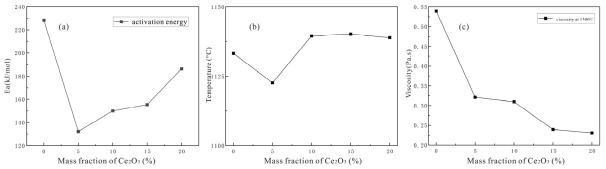
Performance parameters of the mold flux at different Ce_2_O_3_ mass fractions: (**a**) viscosity; (**b**) temperature; (**c**) activation energy.

Figure 19a shows the dependence of melting temperature on Ce_2_O_3_ mass fraction. As Ce_2_O_3_ increased, the melting temperature decreased slightly at first, then increased, and finally approached a stable plateau. The minimum melting temperature (~1122.6 °C) was obtained at 5 wt.% Ce_2_O_3_. When Ce_2_O_3_ exceeded 10 wt.%, the melting temperature remained relatively stable at approximately 1140 °C.

**Figure 20:**
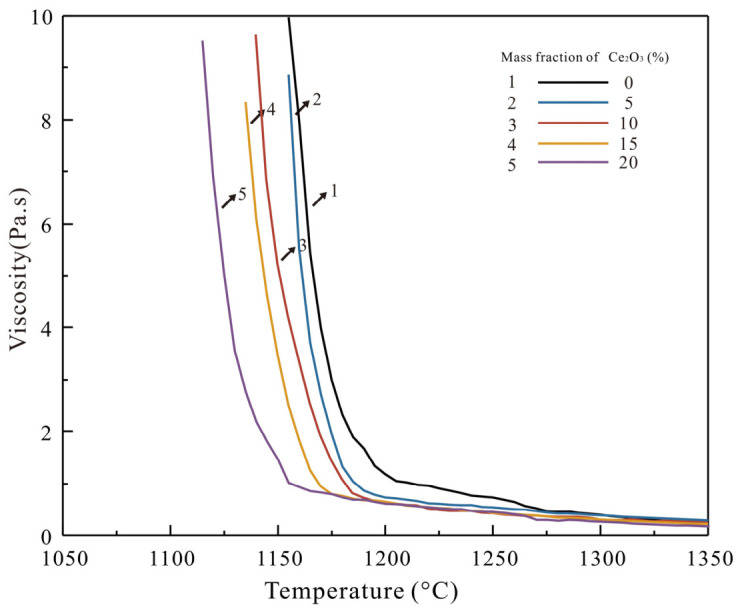
Viscosity–temperature curves of the mold flux at different Ce_2_O_3_ mass fractions.

**Figure 21:**
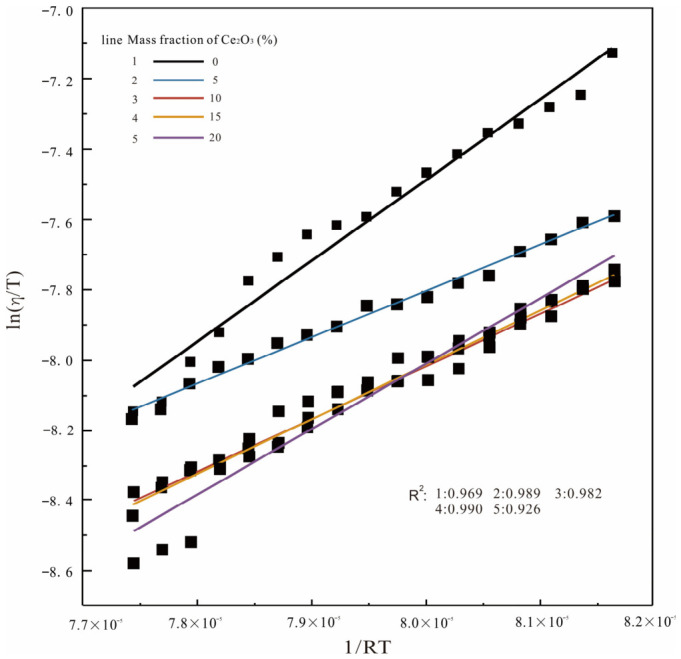
Functional relationship between mold flux viscosity and temperature at different Ce_2_O_3_ mass fractions.

Figure 19 shows the performance parameters of the mold flux at different Ce_2_O_3_ mass fractions. Figure 20 presents the viscosity–temperature curves of the mold flux under different Ce_2_O_3_ mass fractions. Figure 21 shows the fitting results for the relationship between mold flux viscosity and temperature.

The viscosity trends further corroborate the compositional effect. With increasing Ce_2_O_3_ mass fraction, the viscosity–temperature curves consistently decreased with increasing temperature, and the inflection temperature shifted downward, reaching a minimum of 1155 °C at 20 wt.% Ce_2_O_3_. At 1300 °C, viscosity decreased with increasing temperature, and when Ce_2_O_3_ exceeded 5 wt.%, the viscosity remained low at ~0.30 Pa·s. The apparent activation energy for viscous flow exhibited a decrease followed by an increase with temperature, reaching a minimum of 132.2 kJ·mol^−1^ at 5 wt.% Ce_2_O_3_; moreover, it was nearly invariant when Ce_2_O_3_ was within 5–15 wt.%.

Combined with the analysis of melt structure, it can be concluded that when the mass fraction of Ce_2_O_3_ does not exceed 10%, the increase in its mass fraction leads to an increase in O^2−^ ions generated by slag dissociation, and the low-polymerization AlO_4_^5−^ tetrahedra Q^2^ and Q^3^ gradually transform into AlO_6_^9−^ octahedra, thereby disrupting the network structure, reducing the degree of polymerization, and gradually lowering the viscosity. When the mass fraction of Ce_2_O_3_ exceeds 10%, the structural units in the slag tend to reach a stable distribution, the degree of polymerization remains relatively stable, and the viscosity also remains relatively stable.

## 5. Comparison of Physicochemical Properties Between the Novel Mold Flux and the Conventional Mold Flux

### 5.1. Development of the Novel Mold Flux

This work used an industrial mold flux applied in the continuous casting of rare-earth weathering steel at a steel plant as a benchmark and carried out the design and development of a new CaO–Al_2_O_3_-based mold flux.

Table 4 summarizes the composition and primary properties of the reference flux, in which CaO accounts for 30–40 wt.% and SiO_2_ for 25–35 wt.%, and the principal fluxing additives are CaF_2_ and Na_2_O.

The reference flux shows a melting temperature range of 1100–1180 °C and a viscosity of ~0.15–0.25 Pa·s at 1300 °C, which are sufficient to satisfy the baseline processing requirements for continuous casting of rare-earth weathering steel at the initial stage.

Nevertheless, prolonged casting aggravates slag–steel interfacial reactions, which induces substantial variations in flux chemistry and properties and makes it difficult to maintain stable and smooth casting operation.

Guided by the performance targets of the plant-used flux (Table 4) and informed by the foregoing findings, an optimized new mold flux for rare-earth weathering steel casting was formulated, and the main compositional ranges are presented in Table 5.

### 5.2. Comparison of Physicochemical Properties Between the Novel and Conventional Mold Fluxes

**Figure 22:**
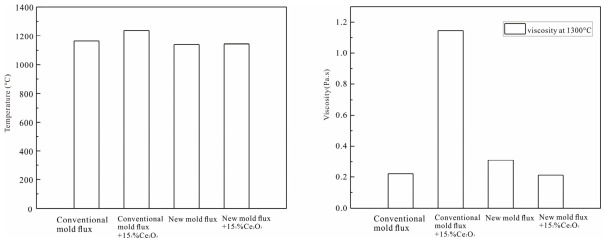
Comparison of the melting temperature of the mold fluxes after absorbing RE oxide.

Figure 22 compares the melting temperatures of the novel CaO–Al_2_O_3_-based mold flux and the conventional mold flux.

As shown in the figure, the melting temperatures are comparable, and the novel flux exhibits a melting temperature of 1140 °C, indicating that its melting behavior is consistent with that of the industrially used flux.

**Figure 23:**
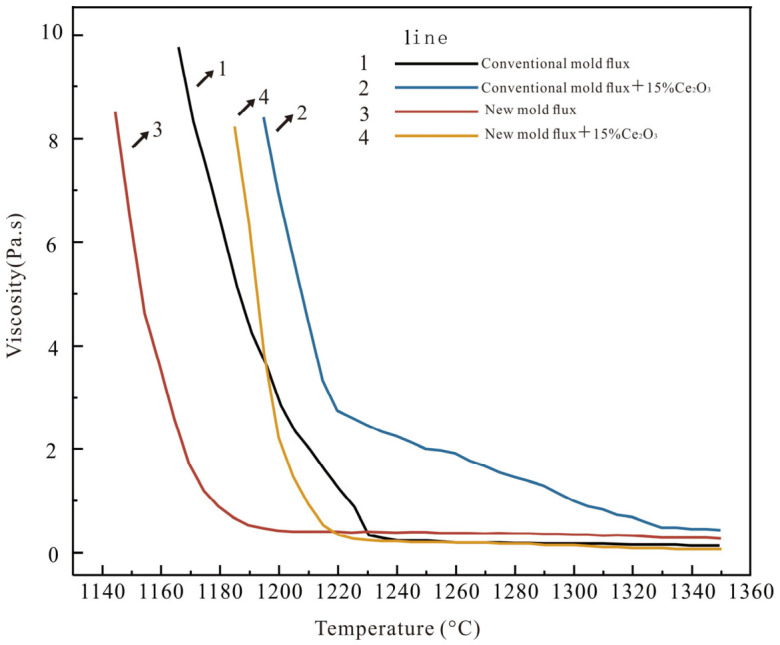
Comparison of the viscous properties of the mold fluxes after absorbing RE oxide.

Figure 23 shows a comparison of the viscosity characteristics of the two mold fluxes.

The novel flux exhibits viscosity characteristics broadly similar to those of the conventional flux, demonstrating good process compatibility in terms of the viscosity–temperature dependence and the workable viscosity window.

**Figure 24:**
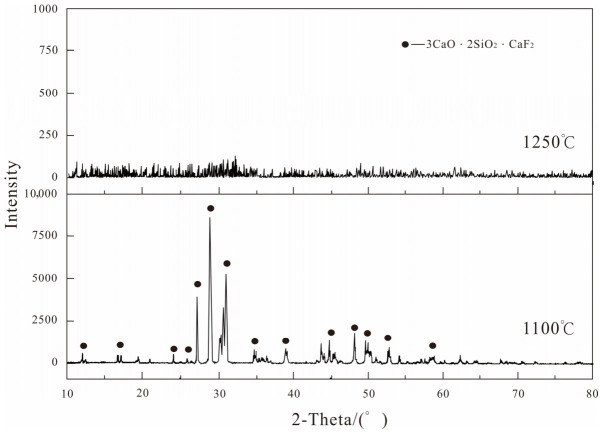
XRD patterns of the conventional mold flux quenched at different temperatures.

To further elucidate the crystallization behavior, XRD analyses were conducted on quenched samples of the conventional flux at the break temperature (1250 °C) and after full crystallization at 1100 °C, as shown in Figure 24.

The results indicate that the conventional flux remains a homogeneous liquid at the break temperature with no detectable crystalline phases, whereas after cooling to 1100 °C and full crystallization, a large amount of cuspidine (3CaO·2SiO_2_·CaF_2_) precipitates.

**Figure 25:**
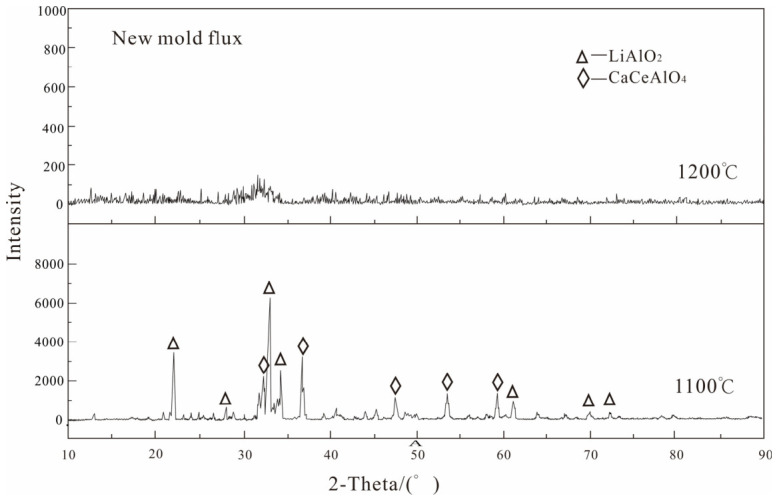
XRD patterns of new mold flux quenched at different temperatures.

Similarly, Figure 25 presents the XRD patterns of quenched samples of the novel flux near the break temperature and after full crystallization at 1100 °C.

The novel flux also behaves as a homogeneous liquid near the break temperature without crystal precipitation; after full crystallization at 1100 °C, two precipitated phases are observed, with LiAlO_2_ as the dominant phase and a minor amount of CaCeAlO_4_.

Overall, the novel CaO–Al_2_O_3_-based flux exhibits a crystallization behavior broadly similar to that of the conventional SPH flux, in that no crystalline phases precipitate above the break temperature and a single liquid phase is maintained, whereas below the break temperature after full crystallization, the main precipitates in the novel flux are LiAlO_2_ and CaCeAlO_4_.

### 5.3. Stability Comparison Between the Novel and Conventional Mold Fluxes

Figure 22 compares the melting-temperature responses of the novel and conventional mold fluxes upon uptake of 15% Ce_2_O_3_.

The conventional silicate-based flux exhibits a pronounced increase in melting temperature of nearly 100 °C after 15% Ce_2_O_3_ uptake; in contrast, the novel flux maintains an almost unchanged melting temperature under the same uptake level, demonstrating excellent melting-temperature stability.

Figure 23 compares the viscosity behavior of the two fluxes following uptake of 15% Ce_2_O_3_.

After Ce_2_O_3_ uptake, the conventional flux exhibits markedly worsened viscosity characteristics, with ~2-fold higher viscosity at 1300 °C (exceeding 1 Pa·s) and an increase of ~100 °C in break temperature.

By comparison, the novel flux undergoes only minor variations in viscosity-related metrics, indicating superior viscosity stability.

**Figure 26:**
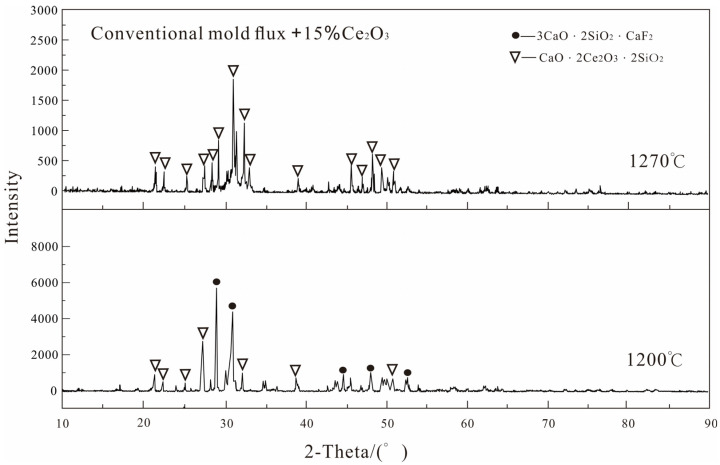
XRD patterns of the conventional mold flux quenched at different temperatures after absorbing RE oxide.

To identify the cause of the deterioration in the conventional flux, quenched samples after 15% Ce_2_O_3_ uptake were examined by XRD near the break temperature (1270 °C) and after complete crystallization at 1200 °C, with the results shown in Figure 26.

The XRD results show extensive precipitation of lath- and block-like CaO·2Ce_2_O_3_·2SiO_2_ at 1270 °C, and after complete crystallization at 1200 °C, a large amount of cuspidine (3CaO·2SiO_2_·CaF_2_) forms in addition to CaO·2Ce_2_O_3_·2SiO_2_.

Extensive formation of high-melting rare-earth silicate phases significantly raises the solid-phase fraction and the degree of melt polymerization, resulting in pronounced viscosity increase and an elevated break temperature.

**Figure 27:**
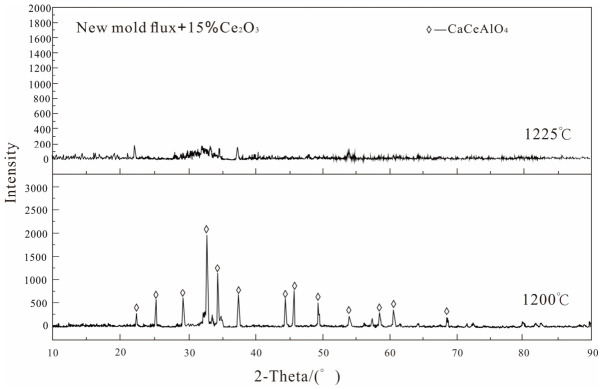
XRD patterns of new mold flux quenched at different temperatures after absorbing RE oxide.

Likewise, Figure 27 shows the XRD patterns of quenched samples of the novel flux after 15% Ce_2_O_3_ uptake near the break temperature (1225 °C) and after complete crystallization at 1200 °C.

The results demonstrate that the novel flux is still a single homogeneous liquid phase near the break temperature with no crystallization detected, while after complete crystallization at 1200 °C, the precipitates become solely CaCeAlO_4_.

In summary, the sharp rises in break temperature and viscosity of the conventional flux upon 15% Ce_2_O_3_ uptake mainly stem from massive precipitation of high-melting rare-earth silicate phases, exemplified by CaO·2Ce_2_O_3_·2SiO_2_.

Over-crystallization elevates the solid fraction of the slag film, potentially causing inadequate lubrication and uneven heat transfer at the mold–strand interface and thus hindering stable continuous casting.

In contrast, even after 15% Ce_2_O_3_ uptake, the novel flux maintains a single liquid phase near the break temperature, better preserving its lubrication function; while precipitation of CaCeAlO_4_ raises the break temperature to a certain extent, the effect is substantially less pronounced than in the conventional flux.

### 5.4. Industrial Implications and Current Limitations

Taken together, the above results indicate that the newly developed CaO–Al_2_O_3_-based mold flux not only exhibits lower reactivity toward rare-earth-bearing molten steel, but also maintains better physicochemical stability after rare-earth oxide uptake than the conventional CaO–SiO_2_-based mold flux. From an industrial perspective, such stability is expected to be beneficial for maintaining lubrication consistency, reducing excessive crystallization of the slag film, and improving the operational stability of continuous casting for rare-earth weathering steels.

In addition to melting temperature, viscosity, and crystallization behavior, the steel–slag interfacial tension is also an important parameter for evaluating mold-flux applicability in continuous casting [23]. Interfacial tension affects slag entrapment tendency, interfacial stability, and lubrication behavior, and therefore has a direct influence on casting smoothness and slab surface quality [24]. In the present work, the improved physicochemical stability of the new mold flux after Ce_2_O_3_ absorption suggests that it may also be favorable for maintaining a more stable steel–slag interface during casting. However, because interfacial tension was not measured in this study, this implication remains qualitative. Further work combining interfacial-tension measurement with interfacial-reaction analysis is therefore needed to provide a more complete evaluation of the practical casting performance of the proposed flux.

Another issue that should be considered for industrial application is the compatibility between the CaO–Al_2_O_3_-based mold flux and refractory materials [25]. Compared with conventional silicate-based mold fluxes, CaO–Al_2_O_3_-rich systems may exhibit different wetting and corrosion behavior toward refractory or ceramic components under prolonged high-temperature service conditions [26]. In the present study, the proposed flux was designed and assessed mainly from the perspectives of slag–metal reactivity and physicochemical stability after rare-earth oxide pickup, whereas its interaction with refractory materials was not investigated. Therefore, although the present results demonstrate that the developed flux is a promising candidate for rare-earth weathering steel casting, its possible influence on refractory service life remains to be clarified. Dedicated compatibility and corrosion tests with representative refractory materials should be carried out before industrial implementation.

Overall, the present study provides a useful design route for developing low-reactivity mold fluxes with improved tolerance to compositional drift during the continuous casting of rare-earth steels. At the same time, the full industrial applicability of the proposed flux still requires further validation through interfacial-property characterization, refractory-compatibility assessment, and pilot-scale or plant-scale casting trials.

## 6. Conclusions

A low-reactivity CaO–Al_2_O_3_-based mold flux for the continuous casting of rare-earth weathering steel was designed and evaluated in the present study through thermodynamic analysis, model calculation, and experimental investigation. The results show that the proposed CaO–Al_2_O_3_–Li_2_O–B_2_O_3_–Ce_2_O_3_ system provides a feasible composition framework for reducing the reactivity of mold fluxes toward rare-earth-bearing molten steel while maintaining acceptable melting, viscosity, and crystallization characteristics.

An activity calculation model for the CaO–Al_2_O_3_–Li_2_O–B_2_O_3_–Ce_2_O_3_ system was established based on the ion and molecule coexistence theory (IMCT). The calculated results indicate that CaO, Li_2_O, and Ce_2_O_3_ exhibit relatively high effective activities, whereas B_2_O_3_ remains at a very low level and Al_2_O_3_ also shows low activity within the present model framework. These results suggest that the newly designed flux system can suppress the contribution of highly reactive components and improve compositional tolerance under rare-earth oxide pickup conditions. However, the present IMCT analysis should be regarded as a semi-quantitative tool for composition screening and relative reactivity evaluation, rather than a direct substitute for experimentally measured absolute thermodynamic activities.

The physicochemical-property measurements show that the viscosity and melting behavior of the proposed flux can be controlled within an appropriate range through compositional optimization. When the Ce_2_O_3_ content exceeded 5 wt.%, the viscosity remained at about 0.30 Pa·s, and when the *w*(CaO)/*w*(Al_2_O_3_) ratio was higher than 1.6, the viscosity stabilized at about 0.25 Pa·s. The minimum melting temperature, approximately 1122.6 °C, was obtained at 5 wt.% Ce_2_O_3_. These results indicate that the proposed system is not only low-reactive in thermodynamic tendency, but also capable of maintaining a practically usable viscosity–melting window for continuous casting operation.

Compared with the conventional CaO–SiO_2_-based mold flux, the newly developed CaO–Al_2_O_3_-based mold flux exhibits similar initial melting and viscosity characteristics but significantly improved stability after rare-earth oxide absorption. After absorbing 15 wt.% Ce_2_O_3_, the conventional mold flux showed a marked increase in melting temperature, viscosity, and break temperature, whereas the newly designed flux remained comparatively stable. This improved stability is associated with the different crystallization evolution of the two systems: the conventional flux tends to form high-melting rare-earth silicate phases, whereas the new flux evolves toward LiAlO_2_- and CaCeAlO_4_-related crystallization products, leading to less severe deterioration in flow and crystallization behavior.

From a practical perspective, the improved stability of the proposed flux after rare-earth oxide absorption is expected to be beneficial for maintaining lubrication consistency, reducing excessive crystallization, and improving casting operability during the continuous casting of rare-earth weathering steels. The present study demonstrates the feasibility of designing a low-reactivity CaO–Al_2_O_3_-based mold flux for rare-earth weathering steel continuous casting; however, several aspects still require further verification before industrial application. In particular, interfacial properties, refractory compatibility, and casting-scale performance were beyond the scope of the present work. Future studies should therefore focus on more comprehensive thermodynamic validation together with plant-relevant evaluation of interfacial behavior, refractory interaction, and slab-quality performance.

## Figures and Tables

**Table 1:** Structural Units in the CaO-Al_2_O_3_-Li_2_O-B_2_O_3_-Ce_2_O_3_ System.

System	Structural Units
	Ca^2+^, Li^+^, Ce^3+^, O^2−^, Al_2_O_3_, B_2_O_3_
Al_2_O_3_-CaO	CaO·Al_2_O_3_, 3CaO·Al_2_O_3_, 12CaO·7Al_2_O_3_, CaO·2Al_2_O_3_, CaO-6Al_2_O_3_
Al_2_O_3_-B_2_O_3_	2Al_2_O_3_·B_2_O_3_, 9Al_2_O_3_ ·2B_2_O_3_
B_2_O_3_-CaO	CaO-2B_2_O_3_, CaO-B_2_O_3_, 2CaO ·B_2_O_3_, 3CaO ·B_2_O_3_
Ce_2_O_3_-Al_2_O_3_	Ce_2_O_3_·Al_2_O_3_, Ce_2_O_3_·11Al_2_O_3_
Li_2_O-Al_2_O_3_	Li_2_O·Al_2_O_3_
Li_2_O-B_2_O_3_	Li_2_O·B_2_O_3_, Li_2_O·2B_2_O_3_, Li_2_O·3B_2_O_3_, Li_2_O·4B_2_O_3_

**Table 2:** The reaction equations involved in the model mainly include.

Project	Structural Units	No	Mass Action Concentration of Structural
Ionic species.	$Ca^{2+}+O^{2-}=\mathrm{CaO}$	(1)	$N_{1}=N_{Ca^{2+}}+N_{O^{2-}}=2x_{1}/\sum X\;\;\;{\;x}_{1}=N_{1}\sum X/2$
	$2Li^{+}+O^{2-}=Li_{2}O$	(2)	$N_{3}=2N_{Li^{+}}+N_{O^{2-}}=3\;x_{3}/\sum X\;\;\;\;x_{3}={\;N}_{3}\sum X/3$
	$2{\mathrm{Ce}}^{3+}+3O^{2-}={\mathrm{Ce}}_{2}O_{3}$	(3)	$N_{5}=2N_{Ce^{3+}}+3N_{O^{2-}}=5\;\;\;x_{5}/\sum X\;{\;x}_{5}=N_{5}\sum X/5$
Molecular species.	$Al_{2}O_{3}$	(4)	$N_{2}={\;x}_{2}/\sum Xx_{2}=N_{2}\sum X$
	$B_{2}O_{3}$	(5)	$N_{4}={\;x}_{4}/\sum Xx_{4}={\;N}_{4}\sum X$
Ion–molecule and molecule–molecule chemical equilibrium reactions	$(Ca^{2+}+O^{2-})+6Al_{2}O_{3}=CaO\cdot$	(6)	$6Al_{2}O_{3}$ $N_{6}=K_{1}N_{1}N_{2}^{6}x_{6}=N_{6}\sum X$
	$(Ca^{2+}+O^{2-})+Al_{2}O=\mathrm{Ca}O\cdot {\mathrm{Al}}_{2}O_{3}$ △G^0^ = −16380 − 37.58T (J/mol)	(7)	$N_{7}=K_{2}N_{1}N_{2}x_{7}=N_{7}\sum X$
	$3(Ca^{2+}+O^{2-})+Al_{2}O_{3}=3CaO\cdot Al_{2}O_{3}$ △G^0^ = −18000 − 18.83T (J/mol)	(8)	$N_{8}=K_{3}N_{1}^{3}N_{2}x_{8}=N_{8}\sum X$
	$12(Ca^{2+}\;+\;O^{2-})\;+\;7Al_{2}O_{3}\;=\;12\mathrm{CaO}\cdot 7Al_{2}O_{3}$ △G^0^ = −12600 − 24.69T (J/mol)	(9)	$N_{9\;}=K_{4}N_{1}^{12}N_{2}^{7}x_{9}={\;N}_{9}\sum X$
	$(Ca^{2+}+\;O^{2-})\;+\;2Al_{2}O_{3\;}=\;\mathrm{CaO}\cdot 2Al_{2}O_{3}$ △G^0^ = −86100 − 205.1T (J/mol)	(10)	$N_{10\;}={\;K}_{5}N_{1}N_{2}^{2}x_{10}=N_{10}\sum X$
	$2Al_{2}O_{3}\;+\;B_{2}O_{3}\;=\;2Al_{2}O_{3}\cdot B_{2}O_{3}$ △G^0^ = −16700 − 25.52T (J/mol)	(11)	$N_{11}={\;K}_{6}N_{2}^{2}N_{4}x_{11}=N_{11}\sum X$
	$9Al_{2}O_{3}\;+\;B_{2}O_{3\;}=\;9Al_{2}O_{3}\cdot B_{2}O_{3}$ △G^0^ = −90958.9 + 36.79T (J/mol)	(12)	$N_{12}={\;K}_{7}N_{2}^{9}N_{4}x_{12\;}={\;N}_{12}\sum X$
	$\left(Ca^{2+}\;+{\;O}^{2-}\right)\;+\;2B_{2}O_{3\;}=\;\mathrm{CaO}\cdot 2B_{2}O_{3}$ △G^0^ = −132385.55 − 28.7T (J/mol)	(13)	$N_{13}={\;K}_{8}N_{1}N_{4}^{2}x_{13}={\;N}_{13}\sum X$
	$(Ca^{2+}+O^{2-})\;+{\;B}_{2}O_{3}\;=\;\mathrm{CaO}\cdot B_{2}O_{3}$ △G^0^ = −109694.16 − 0.67T (J/mol)	(14)	$N_{14}=K_{9}N_{1}N_{4}x_{14\;}=N_{14}\sum X$
	$2(Ca^{2+}+O^{2-})\;+{\;B}_{2}O_{3}\;=\;2\mathrm{CaO}\cdot B_{2}O_{3}$ △G^0^ = −75362.4 − 20.77T (J/mol)	(15)	$N_{15\;}=K_{10}N_{1}^{2}N_{4}x_{15\;}=N_{15}\sum X$
	$3(Ca^{2+}+O^{2-})\;+\;B_{2}O_{3}\;=\;3\mathrm{CaO}\cdot B_{2}O_{3}$ △G^0^ = −108019.44 − 46.56T (J/mol)	(16)	$N_{16}\;=\;K_{11}N_{1}^{3}N_{4}x_{16}\;=\;N_{16}\sum X$
	$(2Ce^{3+}+3O^{2-})\;+\;Al_{2}O_{3}\;=\;Ce_{2}O_{3}\cdot Al_{2}O_{3}$ △G^0^ = −129790.8 − 54.6T J/mol)	(17)	$N_{17\;}={\;K}_{12}N_{2}N_{5}x_{17}={\;N}_{17}\sum X$
	$(2Ce^{3+}+3O^{2-})\;+\;11Al_{2}O_{3\;}=\;Ce_{2}O_{3}\cdot 11Al_{2}O_{3}$ △G^0^ = −60240.99 − 14.19T(J/mol)	(18)	$N_{18}=K_{13}N_{2}^{11}N_{5}x_{18\;}={\;N}_{18}\sum X$
	$(2Li^{+}+O^{2-})\;+\;Al_{2}O_{3}\;=\;Li_{2}O\cdot Al_{2}O_{3}$ △G^0^ = 49331.82 − 80.56T (J/mol)	(19)	$N_{19}=K_{14}N_{2}N_{3}x_{19}=N_{19}\sum X$
	$(2Li^{+}+O^{2-})\;+{\;B}_{2}O_{3}\;=\;Li_{2}O\cdot B_{2}O_{3}$ △G^0^ = −107100 − 10.59T (J/mol)	(20)	$N_{20}=K_{15}N_{3}N_{4}x_{20}={\;N}_{20}\sum X$
	$(2Li^{+}+O^{2-})\;+\;2B_{2}O_{3}\;=\;Li_{2}O\cdot 2B_{2}O_{3}\cdot$ △G^0^ = −134250 − 23.8T (J/mol)	(21)	$N_{21}=K_{16}N_{3}N_{4}^{2}x_{21}={\;N}_{21}\sum X$
	$(2Li^{+}+O^{2-})\;+\;3B_{2}O_{3}=Li_{2}O\cdot 3B_{2}O_{3}$ △G^0^ = −260640 + 66.97T (J/mol)	(22)	$N_{22}=K_{17}N_{3}N_{4}^{3}x_{22}={\;N}_{22}\sum X$
	$(2Li^{+}+O^{2-})\;+\;4B_{2}O_{3}\;=\;Li_{2}O\cdot 4B_{2}O_{3}$ △G^0^ = −368200 + 156.9T (J/mol)	(23)	$N_{23}=K_{23}N_{3}N_{4}^{4}x_{23}={\;N}_{23}\sum X$

**Table 3:** Activities of the components in the CaO-Al_2_O_3_-based mold flux.

Component(s)	Mass Fraction (wt.%)	Component Activity at Different Temperatures				
		1100 °C	1200 °C	1300 °C	1400 °C	1500 °C
CaO	35	0.467723	0.466370	0.464943	0.463335	0.461468
Al_2_O_3_	35	0.000023	0.000041	0.000070	0.000110	0.000165
Li_2_O	15	0.240265	0.243249	0.246438	0.249878	0.253599
Ce_2_O_3_	10	0.073709	0.071970	0.070133	0.068257	0.066393
B_2_O_3_	5	0.000000	0.000000	0.000000	0.000000	0.000000
Li_2_O·Al_2_O_3_		0.178839	0.178281	0.177555	0.176655	0.175579
CaO·Al_2_O_3_		0.000314	0.000531	0.000836	0.001241	0.001753
3CaO·Al_2_O_3_		0.000075	0.000132	0.000214	0.000326	0.000470
Ce_2_O_3_·Al_2_O_3_		0.001280	0.001636	0.002009	0.002387	0.002761
2CaO·B_2_O_3_		0.004143	0.004637	0.005107	0.005552	0.005973
3CaO·B_2_O_3_		0.028500	0.028317	0.028090	0.027831	0.027547

**Table 4:** Composition and properties of the mold flux for rare-earth weathering steel continuous casting.

Mass Fraction %						Properties	
CaO	SiO_2_	Al_2_O_3_	Na_2_O	F	C	Melting temperature/°C	viscosity (1300 °C)/Pa·s
30~40	25~35	5~10	5~15	5~10	4~5	1100~1180	0.15~0.25

**Table 5:** Composition of the new mold flux for rare-earth weathering steel continuous casting (mass fraction)%.

CaO	Al_2_O_3_	Li_2_O	B_2_O_3_	Ce_2_O_3_
43.1~49.2	26.9~30.8	5~10	5~10	0~10

## Data Availability

The original contributions presented in this study are included in the article. Further inquiries can be directed to the corresponding author.
